# Single-Nucleotide Polymorphisms in Genes Maintaining the Stability of Mitochondrial DNA Affect the Occurrence, Onset, Severity and Treatment of Major Depressive Disorder

**DOI:** 10.3390/ijms241914752

**Published:** 2023-09-29

**Authors:** Piotr Czarny, Sylwia Ziółkowska, Łukasz Kołodziej, Cezary Watała, Paulina Wigner-Jeziorska, Katarzyna Bliźniewska-Kowalska, Katarzyna Wachowska, Małgorzata Gałecka, Ewelina Synowiec, Piotr Gałecki, Michał Bijak, Janusz Szemraj, Tomasz Śliwiński

**Affiliations:** 1 Department of Medical Biochemistry, Medical University of Lodz, 92-215 Lodz, Poland; sylwia.ziolkowska@umed.lodz.pl (S.Z.); janusz.szemraj@umed.lodz.pl (J.S.); 2 Laboratory of Medical Genetics, Faculty of Biology and Environmental Protection, University of Lodz, 92-215 Lodz, Poland; lukasz.kolodziej@edu.uni.lodz.pl (Ł.K.);; 3 Department of Haemostatic Disorders, Medical University of Lodz, 92-215 Lodz, Poland; cezary.watala@umed.lodz.pl; 4 Department of General Biochemistry, Faculty of Biology and Environmental Protection, University of Lodz, 90-136 Lodz, Poland; paulina.wigner.jeziorska@biol.uni.lodz; 5 Department of Adult Psychiatry, Medical University of Lodz, 91-229 Lodz, Poland; katarzyna.blizniewska-kowalska@umed.lodz.pl (K.B.-K.); katarzyna.wachowska@umed.lodz.pl (K.W.); piotr.galecki@umed.lodz.pl (P.G.); 6 Department of Psychotherapy, Medical University of Lodz, 91-229 Lodz, Poland; malgorzata.galecka@umed.lodz.pl; 7 Biohazard Prevention Centre, Faculty of Biology and Environmental Protection, University of Lodz, 90-136 Lodz, Poland; michal.bijak@biol.uni.lodz.pl

**Keywords:** oxidative stress, depression, DNA repair, DNA damage, mitochondrial DNA, gene polymorphism, major depressive disorder

## Abstract

One of the key features of major depressive disorder (MDD, depression) is increased oxidative stress manifested by elevated levels of mtROS, a hallmark of mitochondrial dysfunction, which can arise from mitochondrial DNA (mtDNA) damage. Thus, the current study explores possibility that the single-nucleotide polymorphisms (SNPs) of genes encoding the three enzymes that are thought to be implicated in the replication, repair or degradation of mtDNA, i.e., POLG, ENDOG and EXOG, have an impact on the occurrence, onset, severity and treatment of MDD. Five SNPs were selected: *EXOG* c.-188T > G (rs9838614), *EXOG* c.*627G > A (rs1065800), *POLG* c.-1370T > A (rs1054875), *ENDOG* c.-394T > C (rs2977998) and *ENDOG* c.-220C > T (rs2997922), while genotyping was performed on 538 DNA samples (277 cases and 261 controls) using TaqMan probes. All SNPs of *EXOG* and *ENDOG* modulated the risk of depression, but the strongest effect was observed for rs1065800, while rs9838614 and rs2977998 indicate that they might influence the severity of symptoms, and, to a lesser extent, treatment effectiveness. Although the SNP located in *POLG* did not affect occurrence of the disease, the result suggests that it may influence the onset and treatment outcome. These findings further support the hypothesis that mtDNA damage and impairment in its metabolism play a crucial role not only in the development, but also in the treatment of depression.

## 1. Introduction

Major depressive disorder (MDD; depression) is one of the most serious psychiatric conditions and a growing problem worldwide. Estimations indicate that its lifetime prevalence exceeds 15%, while currently as many as 280 million people are affected by it [1,2,3]. One of the key features of the disease is lowering of the mood and anhedonia, i.e., the lack of pleasure in doing activities that used to give enjoyment, both of which have a negative impact on family, social and professional life [2]. Unfortunately, in extreme cases, it can lead to suicide. Apart from this, some researchers point out the lack of cellular and molecular markers and the fact that diagnosis is based only on observations [4]. Another problem is drug-resistant depression—approximately one third of patients do not response to pharmacotherapy, while the effectiveness of the treatment can be evaluated after 6 weeks [5,6]. Thus, there is a necessity for further research that will elucidate these problems. 

Ongoing research revealed several factors that may contribute to disease development, including inflammation, oxidative and nitrosative stress (O&NS), DNA damage, DNA repair and mitochondrial dysfunction, which seem to be interplaying with each other [7,8,9]. However, the exact mechanism remains elusive. In recent years, several studies, including meta-analysis, found increased oxidative DNA damage in various biological materials taken from depressed patients [10,11,12,13,14,15,16,17]. Our research team was one of the first to show that the elevated DNA damage observed in depression is not only the result of increased oxidative stress, but also impairments in nuclear DNA (nDNA) damage repair [18,19]. Furthermore, we showed that single-nucleotide polymorphisms (SNPs) located in genes encoding proteins involved mainly in base excision repair (BER), a main pathway responsible for the repair of oxidative DNA damage, modulate the risk of depression incidence [20,21,22]. Additionally, genotype–phenotype analysis revealed that these SNPs might also affect the efficacy of DNA damage repair, and by this contribute to the elevated DNA damage [19]. 

Increased oxidative stress, including elevated levels of mitochondrial reactive oxygen species (mtROS), associated with depression might imply the presence of mitochondrial dysfunctions [23,24]. Indeed, several papers employing both animal models and clinical observational studies confirmed the presence of numerous abnormalities in the mitochondria of depressed patients [25,26]. One of the working hypotheses is that such abnormalities can arise from mitochondrial genome instability [27,28,29]. Indeed, elevated levels of mitochondrial DNA (mtDNA) deletions, which can arise from single-strand DNA breaks caused by ROS [30], were found in muscle biopsies taken from depressed patients [31]. Further, an increased amount of 8-oxo-7,8-dihydro-2′-deoxyguanosine (8-oxo-dG), an oxidative DNA damage marker, was detected in mtDNA isolated from the patients’ lymphocytes [32]. Our own research also indicated a higher amount of mtDNA damage in peripheral blood mononuclear cells (PBMC) in the course of depression [33]. Another factor, that was broadly studied in the context of depression, was the mtDNA copy number (mtDNAcn), as it can also be used to evaluate the stability of a mitochondrial genome [34,35]. However, due to confounding factors, i.e., episode severity, pharmacotherapy, the type of biological material that was studied or the patient’s naivety, the obtained results were inconclusive and showed no change [33,36,37], increase [38,39,40,41] or decrease [32,42] in mtDNAcn. Thus, it was suggested that circulating cell-free mtDNA (ccf-mtDNA) could be a more suited marker for depression [43], which can also be associated with mitochondrial damage as well as an increased rate of apoptosis and necrosis [44,45,46]. However, further reports, including meta-analysis, also gave inconclusive results [47,48,49,50,51]. Lastly, our own research indicated impairments in mtDNA repair and degradation in depressed patients’ PBMCs after exposure to oxidative stressed induced by hydrogen peroxide [33]. 

The aforementioned studies indicated that the increased oxidative stress observed in depressed patients may arise from the instability of mtDNA. To this end, the present study explores the possible association between the incidence, onset, severity and treatment of depression and the genotypes or alleles of five SNPs: *EXOG* c.-188T > G (rs9838614), *EXOG* c.*627G > A (rs1065800), *POLG* c.-1370T > A (rs1054875), *ENDOG* c.-394T > C (rs2977998) and *ENDOG* c.-220C > T (rs2997922). These polymorphisms are located in genes encoding proteins that are considered to be responsible for maintaining mitochondrial genome integrity, i.e., its replication, repair and degradation. 

## 2. Results

### 2.1. Single-Nucelotide Polymorphisms of Genes Involved in Mitochodrial DNA Metabolism Are Linked to the Incidence of Depression

The distribution of the genotypes and alleles of the studied SNPs in depressed patients and the control group as well as odds ratios (ORs) with corresponding confidence intervals (CIs) are shown in Table 1. Only polymorphisms located in *ENDOG* and *EXOG* modulated the risk of the disease. Precisely, genotype G/G and allele G of both *EXOG* c.-188T > G (rs9838614) and c.*627G > A (rs1065800), and genotype C/C and allele C of *ENDOG* c.-220C > T (rs2997922) decreased, while allele T of *EXOG* c.-188T > G (rs9838614), genotype G/A and allele A of c.*627G > A (rs1065800), genotype T/T of *ENDOG* c.-394T > C (rs2977998) and genotype C/C and allele C of *ENDOG* c.-220C > T (rs2997922) increased the odds ratio of depression occurrence. No significant results were found for *POLG* c.-1370T > A (rs1054875).

### 2.2. Haplotypes of the Studied Single-Nucelotide Polymorphisms Located in ENDOG and EXOG Are Associated with the Incidence of Depression

Since each SNP located in *ENDOG* and *EXOG* genes gave statistically significant results, one could speculate that their haplotypes may also modulate the risk of the disease. To this end, online SHEsisPlus software (http://shesisplus.bio-x.cn/SHEsis.html, accessed on 10 June 2023) [52] was used to test this hypothesis, and the results are presented in Table 2. Analysis revealed that the haplotypes of both genes influenced the incidence of depression: TG and GA of *EXOG* and CC of *ENDOG* had a protective effect, while TT of *EXOG* and CT and TT of *ENDOG* significantly increased the OR of an episode occurrence. 

### 2.3. Single-Nucleotide Polymorphisms of Genes Involved in Mitochondrial DNA Metabolism Are Linked to the Onset of Depression

**Figure 1:**
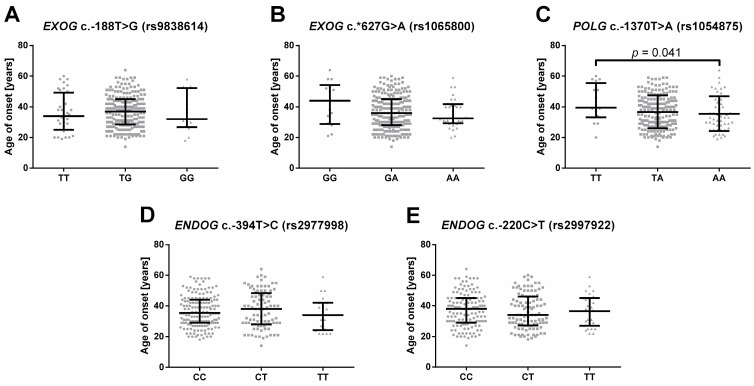
Age of depression onset in patients with different genotypes of (**A**) *EXOG* c.-188T > G (rs9838614), (**B**) *EXOG* c.*627G > A (rs1065800), (**C**) *POLG* c.-1370T > A (rs1054875), (**D**) ENDOG c.-394T > C (rs2977998) and (**E**) ENDOG c.-220C > T (rs2997922). Results are presented as scatter dot plots, horizontal lines denote median, while whiskers represent interquartile range.

The evaluation of whether the studied SNP have an impact on the onset of MDD was performed utilizing two approaches. The first one used age of onset as a continuous variable, which was then compared between patients carrying different genotypes of the studied SNPs. The comparison, shown in Figure 1A–E, revealed that the only statistically significant difference was between the homozygotes of *POLG* c.-1370T > A (rs1054875) (Figure 1C). 

In the second approach, patients were stratified with a cut-off set for the age of the first episode at 35 years, dividing them into two groups: early and late-onset depression. The distribution of the genotypes and alleles of the studied SNP in each of the groups, and their comparison with the control group, are presented in Table 3. Several unique results were obtained only in early onset depression: alleles of *EXOG* c.-188T > G (rs9838614) modulated its risk, while the C/C genotype of *ENDOG* c.-220C > T (rs2997922) had a protective effect. No significant results were found when patients with early and late-onset depression were compared with each other (Supplementary Table S1.). However, it should be mentioned that borderline significance was obtained for alleles of *POLG* c.-1370T > A (rs1054875) (*p* = 0.053).

### 2.4. Single-Nucleotide Polymorphisms of Genes Involved in Mitochondrial DNA Metabolism Are Linked to the Severity of the Depression Episode

**Figure 2:**
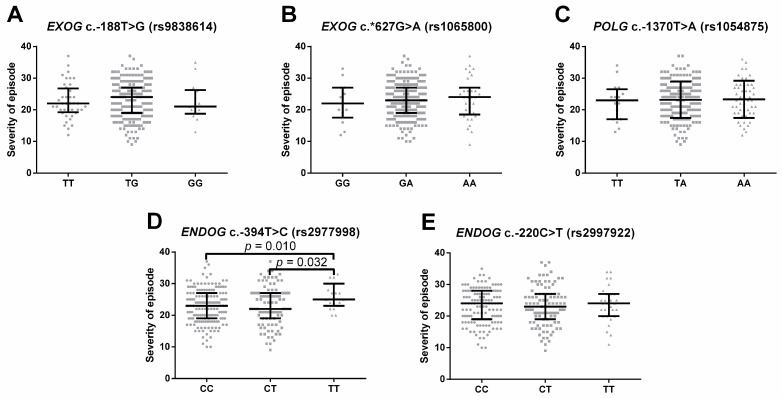
Severity of current episode before treatment measured using the 21-item Hamilton Depression Rating Scale (HAM-D) in depressed patients with different genotypes of (**A**) *EXOG* c.-188T > G (rs9838614), (**B**) *EXOG* c.*627G > A (rs1065800), (**C**) *POLG* c.-1370T > A (rs1054875), (**D**) *ENDOG* c.-394T > C (rs2977998) and (**E**) *ENDOG* c.-220C > T (rs2997922). Results are presented as scatter dot plots, horizontal lines denote median, while whiskers represent interquartile range.

An analogous two-way approach was used to study the impact of the SNPs on the severity of the depression episode that was assessed using the 21-item Hamilton Depression Rating Scale (HAM-D) [53]. Firstly, the HAM-D score was used as continuous data and compared between patients with different genotypes, which is shown in Figure 2A–E. It was revealed that the T/T carriers of *ENDOG* c.-394T > C (rs2977998) had significantly more severe episodes than the heterozygotes and other homozygotes (Figure 2D).

Secondly, patients were stratified using a cut-off set on a HAM-D score of 23, forming groups with severe and moderate symptoms. The distribution of the genotypes and alleles of the studied polymorphism in both groups as well as the comparison with the control group are presented in Table 4. Though alleles of *EXOG* c.-188T > G (rs9838614) modulated the risk of moderate depression only and the T/G genotype of the same SNP increased this risk, the G/G genotype had a protective effect only against severe depression. In the case of *ENDOG* c.-394T > C (rs2977998), significant results were obtained only for patients with a higher HAM-D score: its alleles were modulated, whereas the T/T genotype increased the odds ratio of the disease occurrence. When the patients with severe and moderate symptoms were compared, the heterozygote variant of *EXOG* c.-188T > G (rs9838614) and the T/T genotype of ENDOG c.-394T > C (rs2977998) were significantly more frequent in the former group (Table 5).

### 2.5. Single-Nucleotide Polymorphisms of Genes Involved in Mitochondrial DNA Metabolism Are Associated with the Treatment of Depression

**Figure 3:**
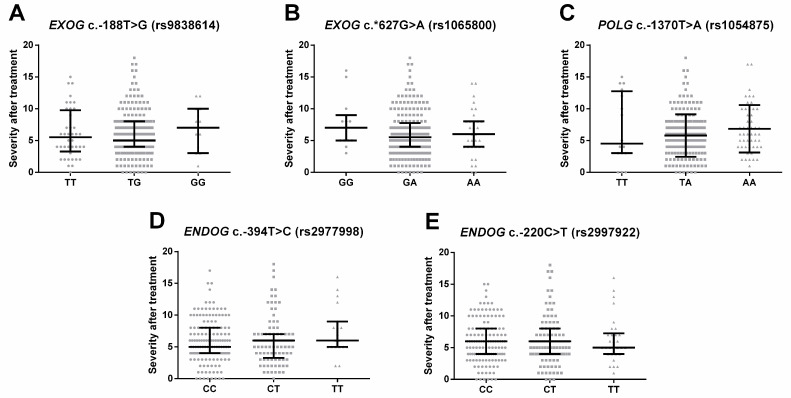
Severity of current episode after treatment measured using the 21-item Hamilton Depression Rating Scale (HAM-D) in depressed patients with different genotypes of (**A**) *EXOG* c.-188T > G (rs9838614), (**B**) *EXOG* c.*627G > A (rs1065800), (**C**) *POLG* c.-1370T > A (rs1054875), (**D**) *ENDOG* c.-394T > C (rs2977998) and (**E**) *ENDOG* c.-220C > T (rs2997922). Results are presented as scatter dot plots, horizontal lines denote median, while whiskers represent interquartile range.

Three analyses were performed to evaluate whether the studied SNP had an impact on the depression treatment. Firstly, the HAM-D after therapy was used as continuous data and was compared between patients with different genotypes. The results, which are shown in Figure 3, displayed no statistically significant differences. 

**Figure 4:**
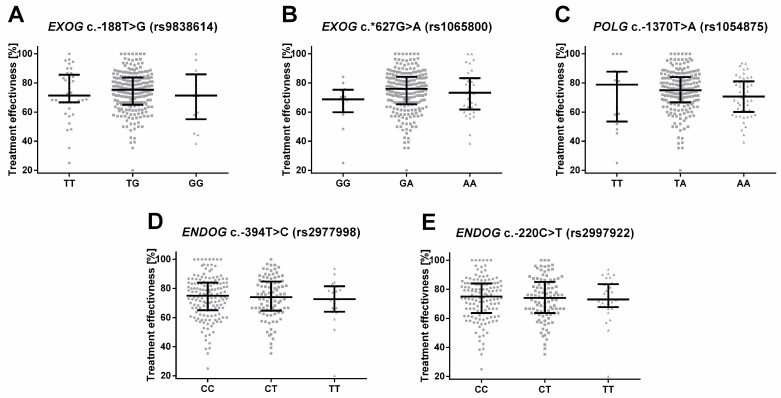
Effectiveness of treatment estimated using the 21-item Hamilton Depression Rating Scale (HAM-D) declined after the treatment and expressed as a percentage in depressed patients with different genotypes of (**A**) *EXOG* c.-188T > G (rs9838614), (**B**) *EXOG* c.*627G > A (rs1065800), (**C**) *POLG* c.-1370T > A (rs1054875), (**D**) *ENDOG* c.-394T > C (rs2977998) and (**E**) *ENDOG* c.-220C > T (rs2997922). Results are presented as scatter dot plots, horizontal lines denote median, while whiskers represent interquartile range.

Secondly, the treatment effectiveness (TE) was calculated using the following equation:

TE = (HAM-D_0_ − HAM-D_E_)/HAM-D_0_ · 100%;

where HAM-D_0_ is the Hamilton score before therapy and HAM-D_E_ is the score after therapy. Then, this variable was compared between depressed patients with different variants of the studied SNPs, which is shown in Figure 4. Although no significant results were found, the distribution of genotype G/G of *EXOG* c.*627G > A (rs1065800) is corelated with borderline significantly less efficient therapy than the heterozygotes (*p* = 0.055).

Lastly, patients were stratified into those that fully recovered (denoted as cured depression) and those that retain some of the symptoms (uncured depression), using the HAM-D score (7 as a cut-off) after therapy. The distribution of genotypes and alleles of the studied SNP in both of the mentioned groups as well as their comparison with the control group are presented in Table 6. Uniquely, only in the case of patients with successful therapy genotype G/G of *EXOG* c.-188T > G (rs9838614) and genotype C/C of *ENDOG* c.-220C > T (rs2997922) decreased, while their alleles modulated the odds ratio of the disease incidence. Moreover, genotype T/A of *POLG* c.-1370T > A (rs1054875) significantly increased the incidence of cured depression. In the case of the patients with less successful therapy, genotype T/T of *ENDOG* c.-394T > C (rs2977998) significantly increased the disease risk. On the other hand, no statistically significant differences in the distribution of the genotypes and the alleles were found between the two groups of patients (Supplementary Table S2).

## 3. Discussion

According to the best of our knowledge, this paper is the first to report that SNPs located in genes encoding proteins maintaining mitochondrial genome integrity, i.e., *EXOG*, *POLG* and *ENDOG*, affect the incidence, onset, severity and treatment of depression. As was mentioned in the introduction, the pathogenesis of depression is closely linked to oxidative stress with characteristic increased production of mtROS [8,23,54]. This feature is regarded as a hallmark of mitochondrial disfunction [24]. Indeed, data in the literature has validated this hypothesis; when using both animal and human studies, the inhibition or disturbed expression of various complexes of the electron transport chain (ETC) [55,56,57,58,59], reduced production of ATP [31,60,61,62] and other abnormalities [25,26] were found. In fact, the pathogenesis of many neurodegenerative diseases, such as Alzheimer’s disease (AD), Parkinson’s disease (PD), Huntington disease (HD), and amyotrophic lateral sclerosis (ALS), and other psychiatric diseases, like schizophrenia (SZ) and bipolar disorder (BD), have been linked with mitochondrial dysfunction [63,64]. This should come as no surprise because of the specificity of central nervous system’s (CNS) metabolism, which makes it remarkably susceptible to oxidative and mitochondrial stress [65,66,67]. 

Mitochondrial DNA contains 16,569 bp and encodes 13 subunits of ETC [68], thus one could speculate that any lesions to it may disturb oxidative phosphorylation and increase the production of ROS [69,70]. To make matters worse, this ROS can further damage mtDNA creating a vicious cycle [71,72]. However, the cells evolved various mechanisms that can prevent this from happening. Firstly, there are mtDNA repair pathways, which have similar components and mechanisms to their nuclear counterparts, except for the lack of nucleotide excision repair (NER) [73]. Moreover, if mtDNA is damaged beyond repair, it can be degraded due to the fact that each mitochondrion contains several copies of its DNA [74,75,76]. The number of mtDNA copies is linked to the cell’s energy consumption and is specific to a given tissue, however, it may vary in different parts of the same tissue, as is the case in the brain [77]. Lastly, the mechanism of mitophagy eliminates dysfunctional mitochondria via autophagy [78,79]. 

The first two of the studied SNPs, c.-188T > G (rs9838614) and c.*627G > A (rs1065800), are located in the upstream region and 3′ untranslated region (3′-UTR) of *EXOG*, respectively. The gene itself is located on chromosome 3 and encodes exo/endonuclease G (EXOG; endonuclease G-like-1), which has both endonuclease activity towards single-stranded DNA (ssDNA) and 5′ to 3′ exonuclease activity [80]. It has been proposed that this enzyme is involved in mitochondrial base excision DNA repair (BER) [81], more specifically, it removes the flap structure created by DNA polymerase during long-path BER (LP-BER) [82]. Interestingly, this subpathway (the other is SP-BER—short-path BER) seems to be predominant in mitochondria, because of the polymerase gamma (Pol γ) weak 5′-deoxyribose phosphate (5′-dRP) lyase activity [73]. Accordingly, knockdown of EXOG using siRNA resulted in increased mtDNA damage caused by the build-up of toxic BER intermediates, mainly single-strand breaks (SSB), which led to mitochondria disfunction and subsequent apoptosis [81,83]. Apart from this, EXOG has been recently found to cooperate with RNase H1 in the removal of the RNA primer during mtDNA replication [84,85]. According to the Variation Viewer of the National Center for Biotechnology Information (NCBI), 12,597 mutations have been located within or near the vicinity of *EXOG* [86]. These include 12,480 single-nucleotide variants present in the Single Nucleotide Polymorphism Database (dbSNP) and 137 various mutations, i.e., copy number variations, insertions, short tandem repeat variations, inversions, mobile element insertions, sequence alteration and tandem duplications, listed in the Database of Genomic Structural Variation (dbVar). The current paper is the first to study *EXOG* in the context of depression. Haplotypes of selected SNPs, as well as individual SNPs, influenced the occurrence of the disease (Table 2), although a particularly strong association was detected for *EXOG* c.*627G > A (rs1065800) (*p* < 0.001). Nonetheless, this SNP does not seems to affect the onset, severity or treatment of the disease. On the other hand, in the case of *EXOG* c.-188T > G (rs9838614) both comparisons between the control group and patients with severe episodes as well as the groups of patients with moderate and severe depression suggest that its heterozygosity was significantly associated with more severe symptoms (Table 4 and Table 5). However, it must be noted that analysis using the HAM-D as continuous data did not give significant results (Figure 1B). Lastly, there are some indications that this SNP may influence the efficiency of the treatment, namely genotype G/G significantly lowered the chance of having treatment that resulted in a HAM-D score equal to or lower than 7, while the alleles modulated this chance (Table 6). Although, this association was observed only when comparison was made to controls and not to uncured patients. As mentioned earlier, the flap structure created during mitochondrial LP-BER can also be processed by FEN1 (Flap structure-specific endonuclease 1) and DNA replication helicase/nuclease 2 (DNA2) [87,88]. However, their knockdown did not result in increased mtDNA damage, mitochondrial dysfunction or apoptosis, as it was when EXOG was knockdown [81], showing EXOG’s greater importance in maintaining mitochondrial genome integrity. Interestingly, our previous study investigated one SNP located in *FEN1*, but it did not influence the incidence of depression [21].

The third of the studied SNPs, c.-1370T > A (rs1054875), is located on chromosome 15 upstream of a gene encoding POLG, a catalytic subunit of the already-mentioned Pol γ [89]. The fully functional protein is composed of one POLG and the dimer of POLG2, encoded by the gene located on chromosome 17 [90]. The dimer enhances polymerase processivity, while the catalytic subunit additionally possesses 3′ to 5′ exonuclease and 5′-dRP lyase activities [91,92]. Pol γ creates a replication complex together with mitochondrial genome maintenance exonuclease 1 (MGME1), twinkle mtDNA helicase (TWNK) and the mitochondrial single-stranded DNA binding protein (mtSSB) [93]. Interestingly, this is the only DNA polymerase found in mitochondria, thus it is involved not only in replication, but also in the repair of mtDNA [94]. Its mutations or reduced activity has been linked with several neurological diseases associated with depletion, deletions or the accumulation of abnormal mtDNA, evidencing its importance in maintaining mitochondrial genome integrity [90,95]. Furthermore, 3′ to 5′ exonuclease activity of Pol γ, which was thought to have only proof-reading functionality during DNA replication, has been recently found to be involved in the degradation of damaged mtDNA [96]. Precisely, elimination of this activity impaired the degradation of linear mtDNA in the modified HEK 293 cell line. Interestingly, the same effect was observed when the inactivation of MGME1 or knockdown of TWNK was performed, implying that whole replication complex is crucial in this phenomenon. To date, 12,313 single-nucleotide variations and 263 other mutations, including copy number variations, insertions, short tandem repeats, variations, inversions, mobile element insertions, complex substitutions, complex chromosomal rearrangement, novel sequence insertion, sequence alteration and tandem duplications, have been found in the gene [86]. Although in the current paper *POLG* c.-1370T > A (rs1054875) did not affect the occurrence or severity of depression (Table 1, Table 4 and Table 5 and Figure 2C), analysis using the age of onset as continuous data revealed significant differences between the homozygotes, namely the A/A genotype carriers had their first episode significantly earlier in their lifespan than the T/T genotype carriers (Figure 1C). On the other hand, stratification of patients into early and late-onset depression did not reveal significant differences in genotypes and alleles’ distribution when compared to the control group (Table 3). However, when both groups of patients were compared, the alleles showed borderline association (*p* = 0.055; Supplementary Table S1), which was in line with the results obtained for continuous data, further suggesting that this SNP may influence onset of the disease (Supplementary Table S1). Secondly, the heterozygosity was significantly associated with an increased chance of treatable depression when compared to the control group (Table 6) and borderline significant when compared to the not fully recovered patients (*p =* 0.063; Supplementary Table S2). Interestingly, in relation to the control group, the A/A genotype reduced the chance of a curable episode, however, this was also of a borderline significance (*p* = 0.062; Table 6). Lastly, the genotype was borderline associated with early onset depression when compared to the controls (*p* = 0.061; Table 3).

The last two SNPs are present upstream of *ENDOG* on chromosome 9. The gene encodes endonuclease G (ENDOG), a paralogue of EXOG with the ability to target both DNA and RNA. ENDOG is one of the seven DNases found in mitochondria, the others being the EXOG studied in this paper, the aforementioned FEN1, DNA2 and MGME1, as well as APEX1 and MRE11, which are involved in DNA repair [93,97]. Its most studied and universally accepted function is the induction of apoptosis in a caspase-independent manner [98]. The enzyme is mainly located in mitochondrial intermembrane space [99] and upon release it is translocated to the nucleus, where it cleaves CG-rich DNA. Apart from this, ENDOG was detected attached to the inner mitochondrial membrane, thus indicating that it might interact with this organelle’s genome and is considered one of the candidates responsible for the degradation of severely damaged mtDNA [99]. Indeed, ENDOG is thought to degrade paternal mtDNA as well as promote paternal mitochondria elimination via autophagy components of maternal origin [100,101]. Due to its RNase activity, it was initially suggested that it also may be involved in the maturation of RNA primers in the initial steps of mtDNA replication [102], however, ENDOG null mice showed no copy number changes or other mtDNA abnormalities [103,104]. Recently, it has been implied that ENDOG may be a crucial player in the activation of autophagy via the suppression of the mTOR pathway with simultaneous stimulation of the DNA damage response via its endonuclease activity [105]. On the one hand, some reports indicated that this enzyme may stimulate the depletion and replication of mtDNA upon the induction of oxidative stress [106]; on the other hand, others proposed replisome as the main mtDNA degradation machinery [96]. Although its role in the mitochondria still remains an open question, ENDOG has been found to play a major role in the pathogenesis of mitochondria-related diseases such as cardiac hypertrophy, Parkinson’s disease and obesity [97,107,108,109]. A total of 2445 mutations have been identified in *ENDOG*, including 2321 single-nucleotide variations and 171 other types of mutations, i.e., copy number variations, insertions, inversions, complex substitutions, sequence alternations and tandem duplications [86]. In the present work, both SNPs located in the gene affected the occurrence of the disease, although in the case of *ENDOG* c.-394T > C (rs2977998) this association (genotype T/T increased risk of the disease) was weaker, even to the point that the result adjusted for sex after bootstrap analysis did not meet statistical significance (Table 1). However, this genotype seems to affect the severity of the episode as well as the treatment efficiency. Firstly, it was significantly associated with an increased chance of a more severe episode, as evidenced by the comparison of the genotypes and alleles distribution between patients with moderate and severe episodes (Table 5). When both groups were compared to the controls, having the TT genotype increased the risk of depression with more severe symptoms only(Table 4). Also, analysis using the HAM-D as continuous data revealed that carriers of the T/T genotype had a significantly higher HAM-D score than both the heterozygotes and the second homozygotes (Figure 1D). Lastly, this genotype increased the chance of an episode that after treatment retained some of the symptoms, while not affecting the incidence of treatable depression (Table 6).

As previously mentioned, the rising number of depression cases, its recuring character and treatment resistance are emerging problems in developed countries. Estimates not only indicate that in one third of cases pharmacotherapy is not viable, but also that treatment evaluation can be performed only after 6 weeks [5,6]. A fast diagnosis and assessment of the therapy using molecular markers could have a huge benefit to the patients, and reduce the sociological and financial burden of the disease. However, the high heterogeneity of the disease makes it very difficult to create a universal panel of such makers. Further, in recent years epigenetics have emerged as the third player, aside from environmental and genetic factors, which can also have a pivotal role in the pathogenesis of depression and the patients’ response to the treatment [110]. The current paper is part of the research trend to seek out the viable panel and overcome the mentioned difficulties. Our results show not only that the studied SNPs are linked to the incidence of depression, but also its treatment efficiency. These, however, must be further explored in other studies, preferably using different ethnic groups, and then validated via meta-analysis. A combination of such research may result in the creation of polygenic risk scores, which have recently started to emerge for depression [111,112,113].

Our work must be perceived through its limitations. Firstly, only five SNPs were selected for this study. This is due to a restriction in amount of samples and the technique used in genotyping, which allows us to determine only one variation per reaction. The criteria used for selection were as follows: a minor allele frequency (MAF) higher than 0.05 (the higher the better), localization in a regulatory or coding region and the availability of probes. However, another open question remains: how do the studied SNPs impact the expression or activity of the proteins, and thus mtDNA integrity? Unfortunately, the literature lacks data that could elucidate this problem, however, localization of these polymorphisms may imply that they could change promotor performance or affect mRNA stability, half-life and degradation [114,115]. As such, this should be also considered as somewhat of a limitation of our work. Another aspect is the relatively small sample size as well as the homogeneity of the studied population, namely, it is exclusively focused on a Polish population. These facts make it difficult to generalize the obtained results for the worldwide population, thus there is a need for our results to be cross-validated with the research recruiting other ethnic groups. 

## 4. Materials and Methods

### 4.1. Characteristics of the Studied Group

There were 538 individuals participating in the study, including 277 patients with MDD and 261 controls. Patients were diagnosed and hospitalized at the Department of Adult Psychiatry of the Medical University of Lodz (Poland). The diagnosis of a depressive episode and recurrent depressive disorder was based on the WHO [116] and ICD-10 (F32.0–7.32.2, F33.0 F33.8) criteria. The detailed characteristics of the groups are presented in Table 7. Before the experiment, a standardized Composite International Diagnostic Interview (CIDI) was used to collect medical history [117], and the severity of the disease symptoms and intensity of the symptoms were assessed using the 21-item Hamilton Depression Rating Scale (HAM-D) [53] and Demyttenaere and De Fruyt [111], respectively. Assessments were conducted before and after antidepressant therapy with selective serotonin reuptake inhibitors (SSRIs). Patients with inflammatory or autoimmune diseases, central nervous system traumas, familial prevalence of mental disorders other than recurrent depressive disorders, and/or the presence of concurrent somatic diseases or axis I and II disorders, other than depressive episodes, were excluded from the study. The study group comprised of unrelated native residents of central Poland and each of the individuals gave their written consent to participate in this study. The protocol was approved by the Bioethics Committee of the Medical University of Lodz (No. RNN/70/14/KE).

### 4.2. DNA Extraction

Genomic DNA was isolated using the Blood Mini Kit (A&A Biotechnology, Gdynia, Poland) from the venous blood of the patients before therapy, and the samples were aliquoted and stored at −20 °C. The purity and quantity of the DNA samples was determined using spectrophotometry.

### 4.3. SNPs Selection and Genotyping

SNPs were selected for the experiments using the public domain of the NCBI database for single-nucleotide polymorphisms (dbSNP), available at http://www.ncbi.nlm.nih.gov/snp (accessed on 22 August 2020) (Bethesda, MD, USA). Genetic variants had to meet the following criteria: a MAF greater than 0.05 (population ID: HapMap-CEU), and a localization in either the coding or regulatory region of the genes. To genotype selected polymorphisms, TaqMan probes (Thermo Fisher Scientific, Waltham, MA, USA) and a 2× Master Mix Takyon for Probe Assay—No ROX (Eurogentec, Liège, Belgium) were used. The experiment was performed using the Mx3005P qPCR System and MxPro QPCR Software (Agilent Technologies, Santa Clara, CA, USA).

### 4.4. Statistical Analysis

The collected data was analyzed in Statistica 12 (Statsoft, Tulsa, OK, USA), SigmaPlot 11.0 (Systat Software Inc., San Jose, CA, USA), Resampling Stats Add-in for Excel v.4 (Arlington, VA, USA) and StudSize3.02 (CreoStat HB, Västra Frölunda, Sweden; used for power analysis). An unconditional multiple logistic regression model was used to obtain the odds ratio (OR) with a 95% confidence interval (95% CI) for the association between case/control and each SNP. ORs were adjusted for gender, and the significant outcomes were further verified with the use of the bootstrap-boosted multiple logistic regression. The goodness of fit of the models’ pointing was estimated with the Hosmer–Lemeshow test. Normality of the studied group was assessed via the Shapiro–Wilk test, while a homogeneity of variance was verified with the Brown–Forsythe test. Then, an unpaired Student’s *t*-test or Mann–Whitney U test was used. Two methods were used to assess the impact of the studied SNPs on the age of onset, severity of the episode and severity after the treatment, both estimated using the HAM-D. The first method analyzed them as a continuous variable, while the second method categorized participants based on cut-offs: (i) the age of 35, representing the transition from young adulthood to middle age; (ii) a score of 23 points in the HAM-D before therapy and (iii) a score of 7 points in the HAM-D after therapy.

## 5. Conclusions

**Figure 5:**
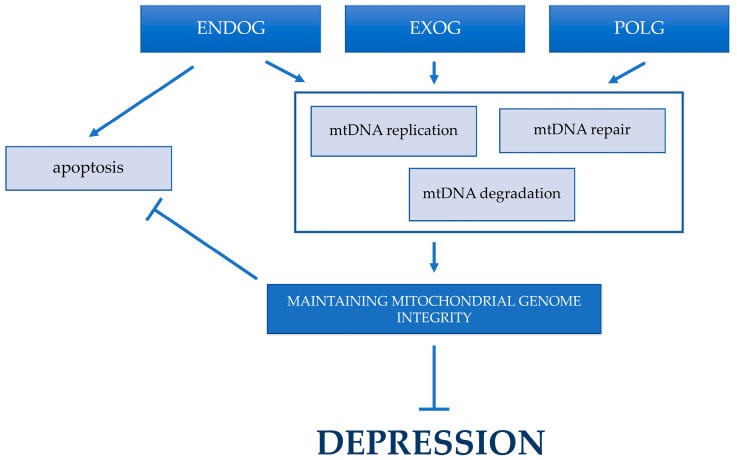
The scheme presenting the association between the studied genes and their role as components maintaining mitochondrial genome integrity in context of depression.

The results presented in the current paper indicate that SNPs located in genes encoding (i) POLG, a subunit of Pol γ involved in the replication, repair and degradation of mtDNA; (ii) ENDOG, an endonuclease with ability to induce apoptosis and autophagy as well as one of the candidates that is speculated to degrade damages mtDNA; and (iii) EXOG, an enzyme involved in mtBER are associated with the occurrence, onset, treatment and severity of depression (Figure 5). They further validate the hypothesis that mitochondrial disfunction caused by mtDNA damage and insufficient DNA repair play a major role in the pathogenesis of this serious mental condition. However, there is still a need for validation of the results on a much larger population, as well as to check the impact of the studied polymorphisms on the expression and activity of the enzymes, and further, to elucidate the causative link between these SNPs and MDD. 

## Figures and Tables

**Table 1:** Distribution of genotypes and alleles of the studied single-nucleotide polymorphisms in the groups of patients with depression and controls without mental disorders.

Genotype /Allele	Control (*n* = 261)		Depression (*n* = 277)		Crude OR (95% CI)	*p*	Adjusted OR (95% CI) *	*p*
	Number	Frequency	Number	Frequency				
*EXOG* c.-188T > G (rs9838614)								
T/T	31	0.119	41	0.148	1.289 (0.781–2.126)	0.320	1.293 (0.783–2.133)	0.315
T/G	195	0.747	222	0.801	1.366 (0.910–2.051)	0.132	1.366 (0.910–2.051)	0.132
G/G	35	0.134	14	0.051	**0.344 (0.180–0.655)** **^&^ 0.338 (0.172–0.664)**	**0.001** **0.002**	**0.342 (0.179–0.652)** **^&^ 0.330 (0.168–0.649)**	**0.001** **0.0015**
***χ*^2^ = 11.672; *p* = 0.003**								
T	257	0.492	304	0.549	**1.677 (1.158–2.428)** **^&^ 1.678 (1.164–2.420)**	**0.006** **0.006**	**1.684 (1.162–2.439)** **^&^ 1.680 (1.151–2.452)**	**0.006** **0.008**
G	265	0.508	250	0.451	**0.596 (0.412–0.863)** **^&^ 0.595 (0.408–0.870)**	**0.006** **0.008**	**0.594 (0.410–0.860)** **^&^ 0.586 (0.399–0.862)**	**0.006** **0.007**
*EXOG* c.*627G > A (rs1065800)								
G/G	75	0.287	14	0.051	**0.132 (0.072–0.241)** **^&^ 0.127 (0.069- 0.237)**	**<0.001** **<0.001**	**0.131 (0.072–0.240)** **^&^ 0.126 (0.066–0.239)**	**<0.001** **<0.001**
G/A	147	0.563	230	0.830	**3.795 (2.549–5.649)** **^&^ 3.841(2.593–5.689)**	**<0.001** **<0.001**	**3.809 (2.558–5.672)** **^&^ 3.836 (2.581–5.701)**	**<0.001** **<0.001**
A/A	39	0.149	33	0.119	0.770 (0.468–1.267)	0.303	0.769 (0.467–1.265)	0.301
***χ*^2^ = 60.160; *p* < 0.001**								
G	297	0.569	258	0.466	**0.486 (0.349–0.676)** **^&^ 0.480 (0.340–0.678)**	**<0.001** **<0.001**	**0.485 (0.349–0.675)** **^&^ 0.479 (0.338–0.679)**	**<0.001** **<0.001**
A	225	0.431	296	0.534	**2.058 (1.480–2.863)** **^&^ 2.081 (1.472–2.942)**	**<0.001** **<0.001**	**2.060 (1.481–2.865)** **^&^ 2.061 (1.471–2.889)**	**<0.001** **<0.0001**
*POLG* c.-1370T > A (rs1054875)								
T/T	24	0.092	18	0.065	0.686 (0.363–1.296)	0.246	0.685 (0.362–1.294)	0.243
T/A	175	0.670	203	0.733	1.348 (0.930–1.953)	0.114	1.350 (0.931–1.956)	0.113
A/A	62	0.238	56	0.202	0.813 (0.540–1.224)	0.322	0.813 (0.540–1.224)	0.321
***χ***^2^ = 2.763; ***p*** = 0.251								
T	223	0.427	239	0.431	1.031 (0.748–1.421)	0.853	1.030 (0.747–1.420)	0.855
A	299	0.573	315	0.569	0.970 (0.704–1.337)	0.853	0.970 (0.704–1.338)	0.855
*ENDOG* c.-394T > C (rs2977998)								
C/C	162	0.621	158	0.570	0.811 (0.575–1.146)	0.235	0.811 (0.575–1.146)	0.235
C/T	90	0.345	98	0.354	1.040 (0.730–1.483)	0.827	1.039 (0.729–1.482)	0.831
T/T	9	0.034	21	0.076	**2.297 (1.032–5.112)** **^&^ 2.365 (1.006–5.563)**	**0.042** **0.049**	**2.305 (1.035–5.131)** **^&^ 2.330 (0.989–5.487)**	**0.041** **0.053**
*χ*^2^ = 4.719; *p* = 0.094								
C	414	0.793	414	0.747	0.774 (0.582–1.029)	0.078	0.773 (0.582–1.028)	0.077
T	108	0.207	140	0.253	1.292 (0.972–1.718)	0.078	1.293 (0.972–1.719)	0.077
*ENDOG* c.-220C > T (rs2997922)								
C/C	155	0.594	140	0.505	**0.699 (0.497–0.983)** **^&^ 0.702 (0.494–0.998)**	**0.040** **0.049**	**0.700 (0.498–0.986)** **^&^ 0.702 (0.502–0.983)**	**0.041** **0.039**
C/T	94	0.360	103	0.372	1.052 (0.740–1.494)	0.779	1.048 (0.737–1.490)	0.795
T/T	12	0.046	34	0.123	**2.903 (1.469–5.739)** **^&^ 3.006 (1.527–5.917)**	**0.002** **0.001**	**2.912 (1.473–5.757)** **^&^ 2.989 (1.452–6.155)**	**0.002** **0.003**
***χ*^2^ = 11.230; *p* = 0.004**								
C	404	0.774	383	0.691	**0.670 (0.513–0.875)** **^&^ 0.666 (0.514–0.864)**	**0.003** **0.002**	**0.671 (0.513–0.876)** **^&^ 0.666 (0.510–0.871)**	**0.003** **0.003**
T	118	0.226	171	0.309	**1.493 (1.142–1.950)** **^&^ 1.493 (1.142–1.952)**	**0.003** **0.003**	**1.491 (1.141–1.948)** **^&^ 1.496 (1.154–1.941)**	**0.003** **0.002**

* results presented as odds ratio (OR) with 95% confidence interval (95%CI), calculated with the aid of multiple logistic regression analysis; crude OR values are adjusted for sex. *p* < 0.05 along with corresponding ORs are in bold. ^&^ the bootstrap-boosted OR values, estimated with the classic resampling procedure with 10,000 iterations, are given for statistical outcomes.

**Table 2:** Distribution of haplotypes of c.-188T > G (rs9838614) and c.*627G > A (rs1065800) of *EXOG*, as well as haplotypes of c.-394T > C (rs2977998) and c.-220C > T (rs2997922) of *ENDOG*, in the group of patients with depression and controls.

Haplotype	Control (*n* = 261)		Depression *(n* = 277)		Crude OR (95% CI)	*p*
	Number	Frequency	Number	Frequency		
*EXOG* c.-188T > G (rs9838614) and c.*627G > A (rs1065800)						
TA	153	0.293	252	0.454	**2.012** **(1.564–2.589)**	**<0.001**
GG	193	0.369	206	0.371	1.009 (0.787–1.292)	0.942
TG	104	0.199	52	0.093	**0.416** **(0.291–0.595)**	**<0.001**
GA	72	0.137	44	0.079	**0.539** **(0.362–0.801)**	**0.001**
*ENDOG* c.-394T > C (rs2977998) and c.-220C > T (rs2997922)						
CC	380	0.727	359	0.648	0.687 (0.530–0.892)	0.005
TC	24	0.045	24	0.043	**0.739** **(0.553–0.987)**	**0.040**
CT	34	0.065	55	0.099	**1.581** **(1.013–2.469)**	**0.046**
TT	84	0.160	116	0.209	**1.380** **(1.012–1.883)**	**0.041**

* results presented as odds ratio (OR) with 95% confidence interval (95%CI). *p* < 0.05 along with corresponding ORs are in bold.

**Table 3:** Distribution of genotypes and alleles of the studied single-nucleotide polymorphisms in the groups of patients with depression that had their first episode before 35 years of age (marked as early onset depression) or after 35 years of age (marked as late-onset depression) and controls without mental disorders.

Genotype /Allele	Control (*n* = 261)		Early Onset Depression (*n* = 129)		Crude OR (95% CI)	*p*	Adjusted OR (95% CI) *	*p*	Late-Onset Depression (*n* = 132)		Crude OR (95% CI)	*p*	Adjusted OR (95% CI) *	*p*
	Number	Frequency	Number	Frequency					Number	Frequency				
*EXOG* c.-188T > G (rs9838614)														
T/T	31	0.119	21	0.163	1.443 (0.792–2.627)	0.231	1.456 (0.799–2.654)	0.220	17	0.129	1.097 (0.583–2.064)	0.775	1.094 (0.581–2.061)	0.780
T/G	195	0.747	100	0.775	1.167 (0.709–1.922)	0.544	1.169 (0.710–1.926)	0.540	109	0.826	1.604 (0.945–2.723)	0.080	1.593 (0.937–2.706)	0.085
G/G	35	0.134	8	0.062	**0.427** **(0.192–0.949)**	**0.037**	**0.420** **(0.189–0.936)**	**0.034**	6	0.045	**0.307** **(0.126–0.751)**	**0.010**	**0.311** **(0.127–0.762)**	**0.011**
					**^&^ 0.403** **(0.171–0.953)**	**0.038**	**^&^ 0.393** **(0.163–0.946)**	**0.037**			**^&^ 0.301** **(0.116–0.778)**	**0.013**	**^&^ 0.299** **(0.112–0.805)**	**0.017**
*χ*^2^ = 5.413; *p* = 0.067									***χ*^2^ = 7.376; *p* = 0.025**					
T	257	0.492	142	0.550	**1.623** **(1.048–2.513)**	**0.030**	**1.641** **(1.059–2.543)**	**0.027**	143	0.542	1.555 (0.993–2.433)	0.054	1.545 (0.986–2.421)	0.058
					**^&^ 1.649** **(1.053–2.583)**	**0.029**	**^&^ 1.644** **(1.068–2.533)**	**0.024**			**^&^ 1.553(1.017–2.370)**	**0.041**	**^&^ 1.547** **(1.001–2.394)**	**0.049**
G	265	0.508	116	0.450	**0.616** **(0.398–0.954)**	**0.030**	**0.610** **(0.393–0.945)**	**0.027**	121	0.458	0.643 (0.411–1.007)	0.054	0.647 (0.413–1.014)	0.058
					**^&^ 0.612** **(0.397–0.942)**	**0.026**	**^&^ 0.604** **(0.389–0.941)**	**0.026**			^&^ 0.635 (0.404–0.999)	0.050	**^&^ 0.639** **(0.422–0.969)**	**0.035**
*EXOG* c.*627G > A (rs1065800)														
G/G	75	0.287	5	0.039	**0.100** **(0.039–0.254)**	**<0.001**	**0.099** **(0.039–0.251)**	**<0.001**	9	0.068	**0.181** **(0.088–0.376)**	**<0.001**	**0.182** **(0.088–0.378)**	**<0.001**
					**^&^ 0.091** **(0.030–0.277)**	**<0.001**	**^&^ 0.083** **(0.028–0.253)**	**<0.001**			**^&^ 0.175** **(0.080–0.381)**	**<0.0001**	**^&^ 0.170** **(0.076–0.377)**	**<0.0001**
G/A	147	0.563	107	0.829	**3.772** **(2.243–6.344)**	**<0.001**	**3.793** **(2.254–6.385)**	**<0.001**	108	0.818	**3.490** **(2.105–5.785)**	**<0.001**	**3.472** **(2.093–5.761)**	**<0.001**
					**^&^ 3.829** **(2.212–6.629)**	**<0.001**	**^&^ 3.838** **(2.182–6.750)**	**<0.001**			**^&^ 3.582** **(2.124–6.038)**	**<0.0001**	**^&^ 3.561** **(2.141–5.923)**	**<0.0001**
A/A	39	0.149	17	0.132	0.864 (0.468–1.595)	0.640	0.867 (0.470–1.603)	0.650	15	0.114	0.730 (0.386–1.379)	0.332	0.737 (0.390–1.393)	0.347
***χ*^2^ = 35.592; *p* < 0.001**									***χ*^2^ = 29.302; *p* < 0.001**					
G	297	0.569	117	0.453	**0.500** **(0.343–0.730)**	**<0.001**	**0.497** **(0.340–0.726)**	**<0.001**	126	0.477	**0.581** **(0.402–0.840)**	**0.004**	**0.580** **(0.401–0.839)**	**0.004**
					**^&^ 0.492** **(0.352–0.689)**	**<0.001**	**^&^ 0.490** **(0.349–0.688)**	**<0.001**			**^&^ 0.580** **(0.418–0.805)**	**0.001**	**^&^ 0.581** **(0.419–0.804)**	**0.001**
A	225	0.431	141	0.547	**2.000** **(1.370–2.920)**	**<0.001**	**2.013** **(1.378–2.942)**	**<0.001**	138	0.523	**1.720** **(1.191–2.485)**	**0.004**	**1.723** **(1.192–2.491)**	**0.004**
					**^&^ 2.023** **(1.464–2.797)**	**<0.001**	**^&^ 2.025** **(1.459–2.812)**	**<0.001**			**^&^ 1.729** **(1.251–2.390)**	**<0.001**	**^&^ 1.739** **(1.249–2.422)**	**0.001**
*POLG* c.-1370T > A (rs1054875)														
T/T	24	0.092	5	0.039	0.398 (0.148–1.069)	0.068	0.398 (0.148–1.069)	0.068	11	0.083	0.898 (0.426–1.894)	0.777	0.901 (0.427–1.901)	0.783
T/A	175	0.670	94	0.729	1.320 (0.828–2.103)	0.243	1.315 (0.825–2.097)	0.250	100	0.758	1.536 (0.959–2.468)	0.076	1.537 (0.956–2.471)	0.076
A/A	62	0.238	30	0.233	0.973 (0.591–1.601)	0.913	0.977 (0.594–1.609)	0.928	21	0.159	0.607 (0.352–1.049)	0.074	0.606 (0.350–1.046)	0.072
*χ*^2^ = 3.718; *p* = 0.156									*χ*^2^ = 3.578; *p* = 0.167					
T	223	0.427	104	0.403	0.844 (0.568–1.254)	0.401	0.841 (0.566–1.251)	0.393	122	0.462	1.279 (0.862–1.899)	0.222	1.283 (0.864–1.905)	0.217
A	299	0.573	154	0.597	1.185 (0.797–1.761)	0.401	1.189 (0.800–1.768)	0.393	142	0.538	0.782 (0.526–1.161)	0.222	0.780 (0.525–1.158)	0.217
*ENDOG* c.-394T > C (rs2977998)														
C/C	162	0.621	76	0.589	0.876 (0.570–1.348)	0.548	0.872 (0.567–1.343)	0.535	76	0.576	0.829 (0.542–1.270)	0.390	0.831 (0.543–1.273)	0.395
C/T	90	0.345	43	0.333	0.950 (0.608–1.484)	0.822	0.948 (0.607–1.482)	0.816	46	0.348	1.016 (0.655–1.577)	0.943	1.013 (0.652–1.573)	0.955
T/T	9	0.034	10	0.078	2.353 (0.932–5.943)	0.070	2.439 (0.961–6.187)	0.061	10	0.076	2.295 (0.909–5.794)	0.079	2.310 (0.914–5.836)	0.077
*χ*^2^ = 3.456; *p* = 0.178									*χ*^2^ = 3.385; *p* = 0.184					
C	414	0.793	195	0.756	0.808 (0.567–1.152)	0.238	0.801 (0.562–1.144)	0.222	198	0.750	0.781 (0.550–1.111)	0.170	0.782 (0.550–1.112)	0.171
T	108	0.207	63	0.244	1.238 (0.868–1.765)	0.238	1.248 (0.874–1.781)	0.222	66	0.250	1.280 (0.900–1.820)	0.170	1.279 (0.899–1.819)	0.171
*ENDOG* c.-220C > T (rs2997922)														
C/C	155	0.594	62	0.481	**0.633** **(0.414–0.968)**	**0.035**	**0.638** **(0.417–0.976)**	**0.038**	69	0.523	0.749 (0.491–1.142)	0.179	0.743 (0.487–1.134)	0.168
					**^&^ 0.635** **(0.413–0.976)**	**0.038**	**^&^ 0.642** **(0.423–0.974)**	**0.037**						
C/T	94	0.360	53	0.411	1.239 (0.804–1.909)	0.331	1.225 (0.794–1.890)	0.359	47	0.356	0.982 (0.635–1.520)	0.936	0.989 (0.639–1.532)	0.961
T/T	12	0.046	14	0.109	**2.526** **(1.133–5.634)**	**0.024**	**2.564** **(1.147–5.727)**	**0.022**	16	0.121	**2.862** **(1.312–6.245)**	**0.008**	**2.869** (**1.314–6.264)**	**0.008**
					**^&^ 2.569** **(1.113–5.930)**	**0.027**	**^&^ 2.566** **(1.098–6.000)**	**0.030**			**^&^ 2.890** **(1.210–6.898)**	**0.017**	**^&^ 2.896** **(1.322–6.340)**	**0.008**
***χ*^2^ = 7.645; *p* = 0.022**									***χ*^2^ = 7.747; *p* = 0.021**					
C	404	0.774	177	0.686	**0.638** **(0.456–0.894)**	**0.009**	**0.640** **(0.457–0.896)**	**0.009**	185	0.701	**0.694** **(0.499–0.965)**	**0.030**	**0.690** **(0.496–0.960)**	**0.028**
					**^&^ 0.635** **(0.448–0.899)**	**0.011**	**^&^ 0.633** **(0.444–0.901)**	**0.011**			**^&^ 0.687** **(0.490–0.964)**	**0.030**	**^&^ 0.690** **(0.491–0.970)**	**0.033**
T	118	0.226	81	0.314	**1.567** **(1.119–2.195)**	**0.009**	**1.563** **(1.116–2.190)**	**0.009**	79	0.299	**1.441** **(1.037–2.003)**	**0.030**	**1.449** **(1.042–2.015)**	**0.028**
					**^&^ 1.568** **(1.112–2.211)**	**0.010**	**^&^ 1.585** **(1.128–2.229)**	**0.008**			**^&^ 1.433** **(1.038–2.004)**	**0.030**	**^&^ 1.446** **(1.038–2.014)**	**0.030**

* results presented as odds ratio (OR) with 95% confidence interval (95%CI), calculated with the aid of multiple logistic regression analysis; crude OR values are adjusted for sex. *p* < 0.05 along with corresponding ORs are in bold. ^&^ the bootstrap-boosted OR values, estimated with the classic resampling procedure with 10,000 iterations, are given for statistical outcomes.

**Table 4:** Distribution of genotypes and alleles of the studied single-nucleotide polymorphisms in the groups of patients suffering from depression that scored less than 23 points in the Hamilton Depression Rating Scale (marked as moderate depression) or more than 23 points in the Hamilton Depression Rating Scale (marked as severe depression) and controls without mental disorders.

Genotype /Allele	Control (*n* = 261)		Moderate Depression (*n* = 130)		Crude OR (95% CI)	*p*	Adjusted OR (95% CI) *	*p*	Severe Depression (*n* = 132)		Crude OR (95% CI)	*p*	Adjusted OR (95% CI) *	*p*
	Number	Frequency	Number	Frequency					Number	Frequency				
*EXOG* c.-188T > G (rs9838614)														
T/T	31	0.119	25	0.192	1.767 (0.994–3.140)	0.053	1.762 (0.991–3.132)	0.054	15	0.114	0.951 (0.494–1.832)	0.881	0.954 (0.495–1.837)	0.887
					^&^ 1.778 (0.963–3.281)	0.065	^&^ 1.778 (0.983–3.218)	0.057						
T/G	195	0.747	96	0.738	0.956 (0.591–1.545)	0.853	0.953 (0.589–1.542)	0.845	112	0.848	**1.895** **(1.092–3.290)**	**0.023**	**1.900** **(1.094–3.300)**	**0.023**
											**^&^ 1.923** **(1.088–3.399)**	**0.025**	**^&^ 1.929** **(1.065–3.494)**	**0.030**
G/G	35	0.134	9	0.069	0.480 (0.223–1.032)	0.060	0.483 (0.225–1.040)	0.063	5	0.038	**0.254** **(0.0972–0.665)**	**0.005**	**0.251** **(0.096–0.659)**	**0.005**
					^&^ 0.455 (0.199–1.043)	0.063	^&^ 0.460 (0.208–1.020)	0.056			**^&^ 0.255** **(0.096–0.677)**	**0.006**	**^&^ 0.253** **(0.096–0.671)**	**0.006**
***χ*^2^ = 6.530; *p* = 0.038**									***χ*^2^ = 9.147; *p* = 0.010**					
T	257	0.492	146	0.562	**1.738** **(1.132–2.669)**	**0.011**	**1.733** **(1.128–2.663)**	**0.012**	142	0.538	1.523 (0.967–2.401)	0.070	1.531 (0.971–2.415)	0.067
					**^&^ 1.726** **(1.136–2.622)**	**0.011**	**^&^ 1.748** **(1.151–2.653)**	**0.009**						
G	265	0.508	114	0.438	**0.575** **(0.375–0.883)**	**0.011**	**0.577** **(0.376–0.886)**	**0.012**	122	0.462	0.656 (0.416–1.035)	0.070	0.653 (0.414–1.030)	0.067
					**^&^ 0.570** **(0.366–0.889)**	**0.013**	**^&^ 0.583** **(0.375–0.907)**	**0.017**						
*EXOG* c.*627G > A (rs1065800)														
G/G	75	0.287	7	0.054	**0.141** **(0.063–0.316)**	**<0.001**	**0.141** **(0.063–0.317)**	**<0.001**	6	0.045	**0.118** **(0.050–0.280)**	**<0.001**	**0.118** **(0.050–0.279)**	**<0.001**
					**^&^ 0.131** **(0.053–0.324)**	**<0.001**	**^&^ 0.131** **(0.053–0.328)**	**<0.001**			**^&^ 0.110** **(0.044–0.276)**	**<0.0001**	**^&^ 0.107** **(0.039–0.294)**	**<0.0001**
G/A	147	0.563	109	0.838	**4.025** **(2.376–6.820)**	**<0.001**	**4.036** **(2.377–6.850)**	**<0.001**	107	0.811	**3.319** **(2.014–5.469)**	**<0.001**	**3.323** **(2.017–5.477)**	**<0.001**
					**^&^ 4.133** **(2.378–7.183)**	**<0.001**	**^&^ 4.130** **(2.404–7.095)**	**<0.001**			**^&^ 3.354** **(2.076–5.419)**	**<0.0001**	**^&^ 3.358** **(2.009–5.646)**	**<0.0001**
A/A	39	0.149	14	0.108	0.687 (0.358–1.317)	0.258	0.691 (0.360–1.325)	0.266	19	0.144	0.957 (0.529–1.732)	0.885	0.958 (0.529–1.734)	0.887
***χ*^2^ = 33.718; *p* < 0.001**									***χ*^2^ = 33.208; *p* = < 0.001**					
G	297	0.569	123	0.473	**0.561** **(0.386–0.816)**	**0.002**	**0.561** **(0.385–0.816)**	**0.002**	119	0.451	**0.497** **(0.342–0.722)**	**<0.001**	**0.496** **(0.342–0.721)**	**<0.001**
					**^&^ 0.555** **(0.401–0.769)**	**0.0004**	**^&^ 0.556** **(0.398–0.778)**	**0.0006**			**^&^ 0.492** **(0.347–0.697)**	**<0.0001**	**^&^ 0.489** **(0.345–0.694)**	**<0.0001**
A	225	0.431	137	0.527	**1.783** **(1.226–2.593)**	**0.002**	**1.783** **(1.226–2.594)**	**0.002**	145	0.549	**2.012** **(1.385–2.922)**	**<0.001**	**2.015** **(1.387–2.927)**	**<0.001**
					**^&^ 1.775** **(1.273–2.475)**	**0.0007**	**^&^ 1.802** **(1.299–2.501)**	**0.0004**			**^&^ 2.038** **(1.467–2.831)**	**<0.0001**	**^&^ 2.033** **(1.476–2.800)**	**<0.0001**
*POLG* c.-1370T > A (rs1054875)														
T/T	24	0.092	8	0.062	0.648 (0.283–1.484)	0.304	0.648 (0.282–1.484)	0.304	8	0.061	0.637 (0.278–1.460)	0.287	0.636 (0.278–1.458)	0.285
T/A	175	0.670	96	0.738	1.388 (0.868–2.217)	0.171	1.391 (0.870–2.222)	0.168	100	0.758	1.536 (0.956–2.468)	0.076	1.537 (0.956–2.470)	0.076
A/A	62	0.238	26	0.200	0.802 (0.479–1.344)	0.403	0.800 (0.478–1.341)	0.398	24	0.182	0.713 (0.421–1.207)	0.208	0.713 (0.421–1.207)	0.208
*χ*^2^ = 2.103; *p* = 0.349									*χ*^2^ = 3.252; *p* = 0.197					
T	223	0.427	112	0.431	1.025 (0.692–1.519)	0.901	1.027 (0.693–1.522)	0.895	116	0.439	1.091 (0.735–1.619)	0.667	1.090 (0.734–1.619)	0.668
A	299	0.573	148	0.569	0.975 (0.658–1.445)	0.901	0.974 (0.657–1.433)	0.895	148	0.561	0.917 (0.617–1.361)	0.667	0.917 (0.618–1.362)	0.668
*ENDOG* c.-394T > C (rs2977998)														
C/C	162	0.621	78	0.600	0.917 (0.596–1.410)	0.692	0.921 (0.598–1.418)	0.708	73	0.553	0.756 (0.495–1.156)	0.197	0.756 (0.495–1.156)	0.197
C/T	90	0.345	47	0.362	1.076 (0.693–1.670)	0.744	1.074 (0.692–1.667)	0.752	45	0.341	0.983 (0.632–1.528)	0.938	0.983 (0.632–1.528)	0.939
T/T	9	0.034	5	0.038	1.120 (0.368–3.412)	0.842	1.100 (0.360–3.365)	0.867	14	0.106	**3.322** **(1.398–7.893)**	**0.007**	**3.321** **(1.397–7.891)**	**0.007**
											**^&^ 3.401** **(1.312–8.816)**	**0.012**	**^&^ 3.429** **(1.351–8.706)**	**<0.01**
*χ*^2^ = 0.168; *p* = 0.919									***χ*^2^ = 8.349; *p* = 0.015**					
C	414	0.793	203	0.781	0.925 (0.638–1.343)	0.683	0.930 (0.640–1.351)	0.704	191	0.723	**0.689** **(0.490–0.969)**	**0.032**	**0.689** **(0.490–0.969)**	**0.032**
											**^&^ 0.696** **(0.498–0.972)**	**0.034**	**^&^ 0.692** **(0.484–0.990)**	**0.044**
T	108	0.207	57	0.219	1.081 (0.745–1.568)	0.683	1.075 (0.740–1.562)	0.704	73	0.277	**1.451** **(1.032–2.040)**	**0.032**	**1.451** **(1.032–2.040)**	**0.032**
											**^&^ 1.446** **(1.026–2.038)**	**0.035**	**^&^ 1.446** **(1.012–2.065)**	**0.043**
*ENDOG* c.-220C > T (rs2997922)														
C/C	155	0.594	65	0.500	0.684 (0.448–1.044)	0.079	0.683 (0.447–1.044)	0.078	66	0.500	0.684 (0.449–1.042)	0.077	0.686 (0.450–1.047)	0.080
C/T	94	0.360	51	0.392	1.147 (0.744–1.769)	0.535	1.152 (0.747–1.779)	0.522	50	0.379	1.083 (0.703–1.670)	0.717	1.079 (0.699–1.665)	0.733
T/T	12	0.046	14	0.108	**2.504** **(1.123–5.584)**	**0.025**	**2.486** **(1.112–5.560)**	**0.027**	16	0.121	**2.862** **(1.312–6.245)**	**0.008**	**2.853** **(1.307–6.229)**	**0.008**
					**^&^ 2.499** **(1.056–5.915)**	**0.041**	**^&^ 2.4478** **(1.028–5.972)**	**0.043**			**^&^ 2.915** **(1.289–6.595)**	**0.010**	**^&^ 2.848** **(1.240–6.543)**	**0.013**
***χ*^2^ = 6.571; *p* = 0.037**									***χ*^2^ = 8.421; *p* = 0.015**					
C	404	0.774	181	0.696	**0.672** **(0.480–0.939)**	**0.020**	**0.673** **(0.481–0.941)**	**0.021**	182	0.689	**0.656** **(0.471–0.912)**	**0.012**	**0.657** **(0.472–0.914)**	**0.013**
					**^&^ 0.671** **(0.474–0.948)**	**0.024**	**^&^ 0.672** **(0.475–0.951)**	**0.025**			**^&^ 0.652** **(0.466–0.913)**	**0.013**	**^&^ 0.655** **(0.474–0.906)**	**0.010**
T	118	0.226	79	0.304	**1.489** **(1.065–2.082)**	**0.020**	**1.486** **(1.063–2.079)**	**0.021**	82	0.311	**1.525** **(1.097–2.122)**	**0.012**	**1.523** **(1.094–2.120)**	**0.013**
					**^&^ 1.483** **(1.052–2.088)**	**0.024**	**^&^ 1.489** **(1.046–2.120)**	**0.027**			**^&^ 1.531** **(1.097–2.137)**	**0.012**	**^&^ 1.532** **(1.093–2.147)**	**0.013**

* results presented as odds ratio (OR) with 95% confidence interval (95%CI), calculated with the aid of multiple logistic regression analysis; crude OR values are adjusted for sex. *p* < 0.05 along with corresponding ORs are in bold. & the bootstrap-boosted OR values, estimated with the classic resampling procedure with 10,000 iterations, are given for statistical outcomes.

**Table 5:** Distribution of genotypes and alleles of the studied single-nucleotide polymorphisms in the groups of patients with depression that scored less than 23 points in the Hamilton Depression Rating Scale (marked as moderate depression) or more than 23 points in the Hamilton Depression Rating Scale (marked as severe depression).

Genotype /Allele	Moderate Depression (*n* = 130)		Severe Depression (*n* = 132)		Crude OR (95% CI)	*p*	Adjusted OR (95% CI) *	*p*
	Number	Frequency	Number	Frequency				
*EXOG* c.-188T > G (rs9838614)								
T/T	25	0.192	15	0.114	0.538 (0.269–1.076)	0.080	0.538 (0.269–1.076)	0.080
					^&^ 0.515 (0.247–1.073)	0.076	0.526 (0.258–1.072)	0.077
T/G	96	0.738	112	0.848	**1.983 (1.071–3.672)**	**0.029**	**1.977 (1.067–3.662)**	**0.030**
					**^&^ 2.065 (1.082–3.940)**	**0.028**	**^&^ 2.019 (1.090–3.736)**	**0.025**
G/G	9	0.069	5	0.038	0.529 (0.172–1.624)	0.266	0.535 (0.174–1.643)	0.275
*χ*^2^ = 4.859; *p* = 0.088								
T	146	0.562	142	0.538	0.785 (0.453–1.361)	0.388	0.783 (0.451–1.357)	0.383
G	114	0.438	122	0.462	1.274 (0.735–2.209)	0.388	1.278 (0.737–2.216)	0.383
*EXOG* c.*627G > A (rs1065800)								
G/G	7	0.054	6	0.045	0.837 (0.273–2.560)	0.755	0.832 (0.272–2.548)	0.747
G/A	109	0.838	107	0.811	0.825 (0.435–1.562)	0.554	0.827 (0.437–1.567)	0.560
A/A	14	0.108	19	0.144	1.393 (0.666–2.912)	0.378	1.391 (0.665–2.909)	0.381
*χ*^2^ = 0.838; *p* = 0.658								
G	123	0.473	119	0.451	0.768 (0.424–1.389)	0.382	0.767 (0.424–1.388)	0.381
A	137	0.527	145	0.549	1.303 (0.720–2.357)	0.382	1.304 (0.720–2.359)	0.381
*POLG* c.-1370T > A (rs1054875)								
T/T	8	0.062	8	0.061	0.984 (0.358–2.705)	0.975	0.974 (0.354–2.682)	0.960
T/A	96	0.738	100	0.758	1.107 (0.633–1.934)	0.722	1.120 (0.640–1.961)	0.691
A/A	26	0.200	24	0.182	0.889 (0.480–1.647)	0.708	0.879 (0.474–1.631)	0.683
*χ*^2^ = 0.146; *p* = 0.929								
T	112	0.431	116	0.439	1.076 (0.653–1.774)	0.773	1.081 (0.656–1.784)	0.759
A	148	0.569	148	0.561	0.929 (0.564–1.532)	0.773	0.925 (0.561–1.525)	0.759
*ENDOG* c.-394T > C (rs2977998)								
C/C	78	0.600	73	0.553	0.825 (0.505–1.347)	0.442	0.830 (0.508–1.358)	0.459
C/T	47	0.362	45	0.341	0.913 (0.550–1.517)	0.727	0.902 (0.542–1.501)	0.691
T/T	5	0.038	14	0.106	**2.966 (1.036–8.490)**	**0.043**	**3.020 (1.053–8.664)**	**0.040**
					^&^ 2.948 (0.993–8.753)	0.051	**^&^ 3.003 (1.007–8.960)**	**0.049**
*χ*^2^ = 4.457; *p* = 0.108								
C	203	0.781	191	0.723	0.746 (0.505–1.102)	0.142	0.748 (0.506–1.105)	0.144
T	57	0.219	73	0.277	1.340 (0.907–1.979)	0.142	1.337 (0.905–1.975)	0.144
*ENDOG* c.-220C > T (rs2997922)								
C/C	65	0.500	66	0.500	1.000 (0.616–1.623)	1.000	1.014 (0.623–1.650)	0.955
C/T	51	0.392	50	0.379	0.945 (0.574–1.554)	0.822	0.859 (0.529–1.394)	0.537
T/T	14	0.108	16	0.121	1.143 (0.533–2.449)	0.731	1.158 (0.539–2.484)	0.707
*χ*^2^ = 0.136; *p* = 0.934								
C	181	0.696	182	0.689	0.971 (0.681–1.385)	0.873	0.976 (0.684–1.393)	0.894
T	79	0.304	82	0.311	1.029 (0.722–1.468)	0.873	1.025 (0.718–1.462)	0.894

* results presented as odds ratio (OR) with 95% confidence interval (95%CI), calculated with the aid of multiple logistic regression analysis; crude OR values are adjusted for sex. *p* < 0.05 along with corresponding ORs are in bold. ^&^ the bootstrap-boosted OR values, estimated with the classic resampling procedure with 10,000 iterations, are given for statistical outcomes.

**Table 6:** Distribution of genotypes and alleles of the studied single-nucleotide polymorphisms in the groups of patients with depression that scored more than 7 points after therapy in HAM-D (marked as cured depression) or more than 7 points after therapy in HAM-D (marked as uncured depression) and controls without mental disorders.

Genotype /Allele	Control (*n* = 261)		Cured Depression (*n* = 167)		Crude OR (95% CI)	*p*	Adjusted OR (95% CI) *	*p*	Uncured Depression (*n* = 95)		Crude OR (95% CI)	*p*	Adjusted OR (95% CI) *	*p*
	Number	Frequency	Number	Frequency					Number	Frequency				
*EXOG* c.-188T > G (rs9838614)														
T/T	31	0.119	26	0.156	1.368 (0.780–2.399)	0.274	1.380 (0.786–2.424)	0.262	14	0.147	1.282 (0.650–2.531)	0.473	1.287 (0.652–2.542)	0.467
T/G	195	0.747	134	0.802	1.374 (0.857–2.204)	0.187	1.372 (0.855–2.200)	0.190	74	0.779	1.193 (0.682–2.086)	0.537	1.176 (0.671–2.060)	0.572
G/G	35	0.134	7	0.042	**0.283** **(0.122–0.652)**	**0.003**	**0.280** **(0.121–0.647)**	**0.003**	7	0.042	0.514 (0.220–1.199)	0.124	0.524 (0.224–1.226)	0.136
					**^&^ 0.269** **(0.110–0.658)**	**0.0041**	**^&^ 0.264** **(0.103–0.679)**	**0.005**						
***χ*^2^ = 10.266; *p* = 0.006**									*χ*^2^ = 2.699; *p* = 0.259					
T	257	0.492	186	0.557	**1.774** **(1.167–2.696)**	**0.007**	**1.790** **(1.176–2.725)**	**0.007**	102	0.537	0.514 (0.220–1.199)	0.124	1.435 (0.886–2.323)	0.142
					**^&^ 1.780** **(1.183–2.680)**	**0.006**	**^&^ 1.817** **(1.218–2.711)**	**0.003**						
G	265	0.508	148	0.443	**0.564** **(0.371–0.857)**	**0.007**	**0.559** **(0.367–0.850)**	**0.007**	88	0.463	0.693 (0.429–1.120)	0.134	0.697 (0.431–1.128)	0.142
					**^&^ 0.559** **(0.369–0.849)**	**0.006**	**^&^ 0.552** **(0.365–0.835)**	**<0.005**						
*EXOG* c.*627G > A (rs1065800)														
G/G	75	0.287	6	0.036	**0.092** **(0.039–0.218)**	**<0.001**	**0.091** **(0.039–0.215)**	**<0.001**	7	0.074	**0.197** **(0.087–0.446)**	**<0.001**	**0.198** **(0.088–0.448)**	**<0.001**
					**^&^ 0.076** **(0.010–0.559)**	**0.011**	**_&_ 0.078** **(0.015–0.394)**	**0.002**			**^&^ 0.183** **(0.077–0.434)**	**0.0001**	**^&^ 0.166** **(0.063–0.432)**	**0.0002**
G/A	147	0.563	143	0.856	**4.621** **(2.812–7.594)**	**<0.001**	**4.720** **(2.864–7.778)**	**<0.001**	73	0.768	**2.573** **(1.506–4.397)**	**<0.001**	**2.567** **(1.502–4.388)**	**<0.001**
					**^&^ 4.720** **(2.845–7.831)**	**<0.0001**	**^&^ 4.843** **(2.914–8.050)**	**<0.0001**			**^&^ 2.607** **(1.519–4.474)**	**<0.0001**	**^&^ 2.632** **(1.518–4.566)**	**<0.0001**
A/A	39	0.149	18	0.108	0.688 (0.379–1.248)	0.218	0.682 (0.376–1.240)	0.210	15	0.158	1.067 (0.558–2.040)	0.844	1.065 (0.557–2.036)	0.850
***χ*^2^ = 48.252; *p* < 0.001**									***χ*^2^ = 18.584; *p* < 0.001**					
G	297	0.569	155	0.464	**0.506** **(0.352–0.727)**	**<0.001**	**0.506** **(0.352–0.727)**	**<0.001**	87	0.458	**0.547(0.368–0.813)**	**0.003**	**0.548(0.369–0.815)**	**0.003**
					**^&^ 0.499** **(0.353–0.705)**	**<0.0001**	**^&^ 0.503** **(0.360–0.703)**	**<0.0001**			**^&^ 0.546** **(0.386–0.771)**	**0.0006**	**^&^ 0.542** **(0.378–0.776)**	**<0.001**
A	225	0.431	179	0.536	**1.977** **(1.375–2.842)**	**<0.001**	**1.977** **(1.375–2.842)**	**<0.001**	103	0.542	**1.829** **(1.230–2.719)**	**0.003**	**1.825** **(1.227–2.714)**	**0.003**
					**^&^ 1.986** **(1.414–2.790)**	**<0.0001**	**^&^ 2.001** **(1.438–2.785)**	**<0.0001**			**^&^ 1.832** **(1.288–2.607)**	**0.0008**	**^&^ 1.867** **(1.315–2.650)**	**<0.0005**
*POLG* c.-1370T > A (rs1054875)														
T/T	24	0.092	9	0.054	0.563 (0.255–1.242)	0.155	0.562 (0.255–1.242)	0.154	7	0.074	0.786 (0.327–1.888)	0.589	0.788 (0.328–1.896)	0.595
T/A	175	0.670	131	0.784	**1.788** **(1.140–2.805)**	**0.011**	**1.785** **(1.137–2.800)**	**0.012**	65	0.684	1.065 (0.643–1.762)	0.807	1.061 (0.641–1.757)	0.818
					**^&^ 1.799** **(1.137–2.845)**	**0.012**	**^&^ 1.796** **(1.149–2.808)**	**0.010**						
A/A	62	0.238	27	0.162	0.619 (0.375–1.022)	0.061	0.621 (0.376–1.025)	0.062	23	0.242	1.025 (0.592–1.776)	0.929	1.028 (0.593–1.781)	0.922
***χ*^2^ = 6.582; *p* = 0.037**									*χ*^2^ = 0.292; *p* = 0.864					
T	223	0.427	149	0.446	1.152 (0.791–1.679)	0.461	1.150 (0.789–1.676)	0.468	79	0.416	0.927 (0.605–1.421)	0.729	0.927 (0.605–1.421)	0.727
A	299	0.573	185	0.554	0.868 (0.596–1.265)	0.461	0.870 (0.597–1.268)	0.468	111	0.584	1.078 (0.704–1.652)	0.729	1.079 (0.704–1.654)	0.727
*ENDOG* c.-394T > C (rs2977998)														
CC	162	0.621	96	0.575	0.826 (0.556–1.227)	0.345	0.826 (0.556–1.227)	0.344	55	0.579	0.840 (0.521–1.355)	0.475	0.848 (0.525–1.368)	0.499
CT	90	0.345	61	0.365	1.093 (0.729–1.640)	0.666	1.090 (0.727–1.636)	0.676	31	0.326	0.920 (0.559–1.516)	0.744	0.908 (0.551–1.499)	0.707
TT	9	0.034	10	0.60	1.783 (0.709–4.486)	0.219	1.824 (0.722–4607)	0.203	9	0.095	**2.930** **(1.127–7.621)**	**0.027**	**2.973** **(1.141–7.747)**	**0.026**
											**^&^ 2.953** **(1.103–7.902)**	**0.031**	**^&^ 2.951** **(1.136–7.666)**	**0.026**
*χ*^2^ = 1.955; *p* = 0.376									*χ*^2^ = 5.270; *p* = 0.072					
C	414	0.793	253	0.757	0.810 (0.581–1.130)	0.215	0.808 (0.579–1.127)	0.209	141	0.742	0.753 (0.511–1.109)	0.150	0.755 (0.512–1.114)	0.157
T	108	0.207	81	0.243	1.234 (0.885–1.722)	0.215	1.238 (0.887–1.728)	0.209	49	0.258	1.329 (0.902–1.958)	0.150	1.324 (0.898–1.952)	0.157
*ENDOG* c.-220C > T (rs2997922)														
C/C	155	0.594	81	0.485	**0.644** **(0.436–0.953)**	**0.028**	**0.645** **(0.436–0.954)**	**0.028**	62	0.481	0.760 (0.474–1.219)	0.255	0.749 (0.466–1.203)	0.232
					**^&^ 0.643** **(0.436–0.947)**	**0.025**	**^&^ 0.648** **(0.438–0.935)**	**0.021**						
C/T	94	0.360	67	0.401	1.190 (0.798–1.775)	0.393	1.183 (0.793–1.767)	0.410	53	0.411	0.990 (0.607–1.616)	0.969	0.996 (0.610–1.626)	0.987
T/T	12	0.046	19	0.114	**2.664** **(1.257–5.644)**	**0.011**	**2.748** **(1.290–5.855)**	**0.009**	14	0.109	**2.717** **(1.156–6.387)**	**0.022**	**2.830** **(1.196–6.694)**	**0.018**
					**^&^ 2.675** **(1.210–5.911)**	**0.015**	**^&^ 2.858** **(1.243–6.571)**	**0.013**			**^&^ 2.790(1.138–6.840)**	**0.025**	**^&^ 2.899(1.133–7.420)**	**0.026**
***χ*^2^ = 9.106; *p* = 0.011**									*χ*^2^ = 5.807; *p* = 0.055					
C	404	0.774	229	0.686	**0.641** **(0.470–0.874)**	**0.005**	**0.639** **(0.469–0.872)**	**0.005**	134	0.705	0.704 (0.486–1.021)	0.064	0.692 (0.476–1.006)	0.054
					**^&^ 0.636** **(0.467–0.867)**	**0.004**	**^&^ 0.634** **(0.462–0.869)**	**<0.005**			^&^ 0.707 (0.497–1.004)	0.052	^&^ 0.687 (0.466–1.014)	0.059
T	118	0.226	105	0.314	**1.560** **(1.144–2.126)**	**0.005**	**1.564** **(1.147–2.132)**	**0.005**	56	0.295	1.420 (0.980–2.057)	0.064	1.444 (0.994–2.099)	0.054
					**^&^ 1.567** **(1.139–2.156)**	**0.006**	**^&^ 1.578** **(1.166–2.137)**	**0.003**			**^&^ 1.447** **(1.011–2.070)**	**0.043**	**^&^ 1.445** **(1.002–2.084)**	**0.049**

* results presented as odds ratio (OR) with 95% confidence interval (95%CI), calculated with the aid of multiple logistic regression analysis; crude OR values are adjusted for sex. *p* < 0.05 along with corresponding ORs are in bold. ^&^ the bootstrap-boosted OR values, estimated with the classic resampling procedure with 10,000 iterations, are given for statistical outcomes.

**Table 7:** The detailed characteristics of patients who qualified for the study.

Depression Severity (HAM-D Range of Scores)	Percentage of Patients Before Treatment	Percentage of Patients After Treatment
None (0–7)	0%	72.52% *
Mild (8–16)	13.74%	26.34% *
Moderate (17–23)	35.88%	1.15% *
Severe (≥24)	50.38%	1.15% *
Mean age of patients ± SD		49.40 ± 10.36 ^#^
Mean age of controls ± SD		53.88 ± 12.39
Gender (male/female) of patients		132/130
Gender (male/female) of controls		132/129 ^&^
Duration of disease from the first episode		Percentage of patients
0–10 years		55.91%
11–20 years		18.90%
21–30 years		15.75%
31–40 years		9.06%
≥41 years		0.39%
Number of episodes		Percentage of patients
1		14.98%
2		30.77%
3		28.74%
4		17.41%
5		4.45%
≥6		3.64%

Significance of comparisons estimated with the Yates-corrected chi^2^ test or the Fisher exact test. * *p* < 0.001; ^#^ *p* = 0.314 vs. controls; ^&^ *p* = 0,810 vs. patients.

## Data Availability

Additional data can be requested via e-mail from the corresponding authors.

## Supplementary Materials

The following supporting information can be downloaded at https://www.mdpi.com/article/10.3390/ijms241914752/s1.
