# Supplementary material for: Single-Nucleotide Polymorphisms in Genes Maintaining the Stability of Mitochondrial DNA Affect the Occurrence, Onset, Severity and Treatment of Major Depressive Disorder
Source: Int J Mol Sci. 2023 Sep 29;24(19):14752. doi: 10.3390/ijms241914752 (PMC10573273; doi:10.3390/ijms241914752)
Supplement: Supplementary file 1 [file ijms-24-14752-s001.zip › ijms-2618090-supplementary.pdf]

**Table S1**. Distribution of genotypes and alleles of the studied single-nucleotide polymorphisms, and OR with 95 % CI in group of depressed patients that had their first episode at or after 35 years of age (marked as late onset depression) and group of depressed patients that had their first episode before 35 years of age (marked as early onset depression).

| Genotype /Allele | Late onset depression (n = 132) | | Early onset depression (n = 129) | | Crude OR (95% CI) | *p* | Adjusted OR (95% CI)* | *p* |
|---|---|---|---|---|---|---|---|---|
| | Number | Frequency | Number | Frequency | | | | |
| *EXOG* c.-188T>G (rs9838614) | | | | | | | | |
| T/T | 17 | 0,129 | 21 | 0,163 | 1,315 (0,659-2,626) | 0,437 | 1,309 (0,654-2,620) | 0,446 |
| T/G | 109 | 0,826 | 100 | 0,775 | 0,728 (0,395-1,340) | 0,308 | 0,723 (0,392-1,335) | 0,300 |
| G/G | 6 | 0,045 | 8 | 0,062 | 1,388 (0,468-4,119) | 0,554 | 1,432 (0,480-4,267) | 0,520 |
| $\chi^2$ = 1,060; *p* = 0.589 | | | | | | | | |
| T | 143 | 0,542 | 142 | 0,550 | 1,096 (0,628-1,911) | 0,747 | 1,084 (0,620-1,894) | 0,777 |
| G | 121 | 0,458 | 116 | 0,450 | 0,913 (0,523-1,591) | 0,747 | 0,923 (0,528-1,612) | 0,777 |
| *EXOG* c.*627G>A (rs1065800) | | | | | | | | |
| G/G | 9 | 0,068 | 5 | 0,039 | 0,551 (0,180-1,691) | 0,298 | 0,551 (0,179-1,696) | 0,299 |
| G/A | 108 | 0,818 | 107 | 0,829 | 1,081 (0,571-2,044) | 0,811 | 1,077 (0,568-2,042) | 0,819 |
| A/A | 15 | 0,114 | 17 | 0,132 | 1,184 (0,564-2,484) | 0,655 | 1,189 (0,565-2,502) | 0,647 |
| $\chi^2$ = 1,238; *p* = 0.538 | | | | | | | | |
| G | 126 | 0,477 | 117 | 0,453 | 0,757 (0,419-1,366) | 0,355 | 0,754 (0,417-1,365) | 0,351 |
| A | 138 | 0,523 | 141 | 0,547 | 1,322 (0,732-2,387) | 0,355 | 1,326 (0,733-2,400) | 0,351 |
| *POLG* c.-1370T>A (rs1054875) | | | | | | | | |
| T/T | 11 | 0,083 | 5 | 0,039 | 0,444 (0,150-1,314) | 0,142 | 0,433 (0,145-1,288) | 0,132 |
| T/A | 100 | 0,758 | 94 | 0,729 | 0,859 (0,493-1,498) | 0,593 | 0,874 (0,500-1,528) | 0,638 |
| A/A | 21 | 0,159 | 30 | 0,233 | 1,602 (0,862-2,977) | 0,136 | 1,582 (0,849-2,946) | 0,148 |
| $\chi^2$ = 3,990; *p* = 0.136 | | | | | | | | |
| T | 122 | 0,462 | 104 | 0,403 | 0,604 (0,363-1,007) | 0,053 | 0,606 (0,364-1,011) | 0,055 |
| A | 142 | 0,538 | 154 | 0,597 | 1,654 (0,993-2,756) | 0,053 | 1,649 (0,989-2,750) | 0,055 |
| *ENDOG* c.-394T>C (rs2977998) | | | | | | | | |

| | | | | | | | | |
|---|---|---|---|---|---|---|---|---|
| CC | 76 | 0,576 | 76 | 0,589 | 1,057 (0,646-1,728) | 0,826 | 1,082 (0,659-1,775) | 0,756 |
| CT | 46 | 0,348 | 43 | 0,333 | 0,935 (0,560-1,560) | 0,796 | 0,905 (0,540-1,516) | 0,704 |
| TT | 10 | 0,076 | 10 | 0,078 | 1,025 (0,412-2,552) | 0,957 | 1,047 (0,419-2,616) | 0,921 |
| | | | | $\chi^2$ = 0,666; $p$ = 0.967 | | | | |
| C | 198 | 0,750 | 195 | 0,756 | 1,029 (0,702-1,508) | 0,882 | 1,040 (0,708-1,527) | 0,841 |
| T | 66 | 0,250 | 63 | 0,244 | 0,972 (0,663-1,424) | 0,882 | 0,962 (0,655-1,412) | 0,841 |
| *ENDOG* c.-220C>T (rs2997922) | | | | | | | | |
| C/C | 69 | 0,523 | 62 | 0,481 | 0,845 (0,520-1,373) | 0,496 | 0,872 (0,534-1,422) | 0,582 |
| C/T | 47 | 0,356 | 53 | 0,411 | 1,261 (0,765-2,079) | 0,363 | 1,214 (0,733-2,012) | 0,451 |
| T/T | 16 | 0,121 | 14 | 0,109 | 0,883 (0,412-1,891) | 0,748 | 0,896 (0,417-1,925) | 0,778 |
| | | | | $\chi^2$ = 0,833; $p$ = 0.659 | | | | |
| C | 185 | 0,701 | 177 | 0,686 | 0,939 (0,658-1,339) | 0,728 | 0,952 (0,666-1,361) | 0,788 |
| T | 79 | 0,299 | 81 | 0,314 | 1,065 (0,747-1,519 | 0,728 | 1,050 (0,735-1,501) | 0,788 |

* OR adjusted for sex.

$p < 0.05$ along with corresponding ORs are in bold

**Table S2**. Distribution of genotypes and alleles of the studied single-nucleotide polymorphisms, and OR with 95 % CI in patients with depression that scored more than 7 point after therapy in HAM-D (marked as cured depression) and more than 7 point after therapy in HAM-D (marked as not cured depression).

| Genotype /Allele | Not cured depression (n = 95) | | Cured depression (n = 167) | | Crude OR (95% CI) | $p$ | Adjusted OR (95% CI)* | $p$ |
|---|---|---|---|---|---|---|---|---|
| | Number | Frequency | Number | Frequency | | | | |
| *EXOG* c.-188T>G (rs9838614) | | | | | | | | |
| T/T | 14 | 0.147 | 21 | 0,163 | 0,937 (0,463-1,897) | 0,857 | 0,936 (0,462-1,897) | 0,854 |
| T/G | 74 | 0.779 | 100 | 0,775 | 0,868 (0,469-1,607) | 0,203 | 0,874 (0,471-1,620) | 0,668 |
| G/G | 7 | 0.042 | 8 | 0,062 | 1,818 (0,618-5,351) | 0,278 | 1,787 (0,606-5,271) | 0,293 |
| | | | | $\chi^2$ = 1,213; $p$ = 0.545 | | | | |
| T | 102 | 0.537 | 142 | 0,550 | 0,814 (0,458-1,445) | 0,494 | 0,817 (0,460-1,452) | 0,491 |

| | | | | | | | | |
|---|---|---|---|---|---|---|---|---|
| G | 88 | 0.463 | 116 | 0,450 | 1,229 (0,692-2,182) | 0,482 | 1,224 (0,689-2,174) | 0,491 |
| *EXOG* c.*627G>A (rs1065800) | | | | | | | | |
| G/G | 7 | 0.074 | 6 | 0,036 | 2,134 (0,696-6,548) | 0,185 | 2,162 (0,703-6,651) | 0,179 |
| G/A | 73 | 0.768 | 143 | 0,856 | 0,557 (0,293-1,060) | 0,075 | 0,552 (0,290-1,053) | 0,071 |
| A/A | 15 | 0.158 | 18 | 0,108 | 1,552 (0,743-3,244) | 0,242 | 1,560 (0,745-3,266) | 0,238 |
| | | | | $\chi^2$ = 3,514; $p$ = 0.173 | | | | |
| G | 87 | 0.458 | 155 | 0,464 | 0,930 (0,505-1,712) | 0,816 | 0,930 (0,504-1,715) | 0,816 |
| A | 103 | 0.542 | 179 | 0,536 | 1,075 (0,584-1,980) | 0,816 | 1,075 (0,583-1,983) | 0,816 |
| *POLG* c.-1370T>A (rs1054875) | | | | | | | | |
| T/T | 7 | 0.074 | 9 | 0,054 | 1,396 (0,503-3,879) | 0,522 | 1,421 (0,510-3,959) | 0,501 |
| T/A | 65 | 0.684 | 131 | 0,784 | 0,595 (0,337-1,051) | 0,074 | 0,581 (0,328-1,029) | 0,063 |
| A/A | 23 | 0.242 | 27 | 0,162 | 1,656 (0,887-3,093) | 0,113 | 1,693 (0,904-3,172) | 0,100 |
| | | | | $\chi^2$ = 3,254; $p$ = 0,197 | | | | |
| T | 79 | 0.416 | 149 | 0,446 | 0,773 (0,459-1,300) | 0,331 | 0,765 (0,454-1,290) | 0,315 |
| A | 111 | 0.584 | 185 | 0,554 | 1,294 (0,769-2,176) | 0,331 | 1,307 (0,775-2,204) | 0,315 |
| *ENDOG* c.-394T>C (rs2977998) | | | | | | | | |
| CC | 55 | 0.579 | 96 | 0,575 | 1,017 (0,611-1,693) | 0,949 | 1,005 (0,602-1,675) | 0,986 |
| CT | 31 | 0.326 | 61 | 0,365 | 0,842 (0,494-1,433) | 0,526 | 0,857 (0,503-1,463) | 0,573 |
| TT | 9 | 0.095 | 10 | 0,60 | 1,643 (0,643-4,198) | 0,300 | 1,610 (0,629-4,125) | 0,321 |
| | | | | $\chi^2$ = 1,278; $p$ = 0.528 | | | | |
| C | 141 | 0.742 | 253 | 0,757 | 0,925 (0,621-1,379) | 0,703 | 0,922 (0,619-1,374) | 0,690 |
| T | 49 | 0.258 | 81 | 0,243 | 1,081 (0,725-1,610) | 0,703 | 1,085 (0,728-1,617) | 0,690 |
| *ENDOG* c.-220C>T (rs2997922) | | | | | | | | |
| C/C | 50 | 0.526 | 81 | 0,485 | 1,180 (0,712-1,953) | 0,521 | 1,154 (0,695-1,917) | 0,579 |
| C/T | 34 | 0.358 | 67 | 0,401 | 0,832 (0,494-1,401) | 0,489 | 0,858 (0,507-1,451) | 0,567 |
| T/T | 11 | 0.116 | 19 | 0,114 | 1,020 (0,463-2,246) | 0,961 | 1,000 (0,453-2,207) | 0,999 |
| | | | | $\chi^2$ = 0,503; $p$ = 0,778 | | | | |
| C | 134 | 0.705 | 229 | 0,686 | 1,088 (0,751-1,578) | 0,654 | 1,080 (0,745-1,566) | 0,685 |
| T | 56 | 0.295 | 105 | 0,314 | 0,919 (0,634-1,332) | 0,654 | 0,926 (0,639-1,343) | 0,685 |

* OR adjusted for sex.

$p < 0.05$ along with corresponding ORs are in bold
